# Destruction of Mycotoxins in Poultry Waste under Anaerobic Conditions within Methanogenesis Catalyzed by Artificial Microbial Consortia

**DOI:** 10.3390/toxins15030205

**Published:** 2023-03-07

**Authors:** Elena Efremenko, Olga Senko, Olga Maslova, Ilya Lyagin, Aysel Aslanli, Nikolay Stepanov

**Affiliations:** Faculty of Chemistry, Lomonosov Moscow State University, Lenin Hills 1/3, 119991 Moscow, Russia

**Keywords:** mycotoxins, bird droppings, alkaline pretreatment, methanogenesis, enzymatic destruction, synthetic consortia

## Abstract

To reduce the toxicity of modern feeds polluted by mycotoxins, various sorbents are added to them when feeding animals. A part of the mycotoxins is excreted from the body of animals with these sorbents and remains in the manure. As a result, bulk animal wastes containing mixtures of mycotoxins are formed. It is known that it is partially possible to decrease the initial concentration of mycotoxins in the process of anaerobic digestion (AD) of contaminated methanogenic substrates. The aim of this review was to analyze the recent results in destruction of mycotoxins under the action of enzymes present in cells of anaerobic consortia catalyzing methanogenesis of wastes. The possible improvement of the functioning of the anaerobic artificial consortia during detoxification of mycotoxins in the bird droppings is discussed. Particular attention was paid to the possibility of effective functioning of microbial enzymes that catalyze the detoxification of mycotoxins, both at the stage of preparation of poultry manure for methanogenesis and directly in the anaerobic process itself. The sorbents with mycotoxins which appeared in the poultry wastes composed one of the topics of interest in this review. The preliminary alkaline treatment of poultry excreta before processing in AD was considered from the standpoint of effectively reducing the concentrations of mycotoxins in the waste.

## 1. Introduction

Today, the attention of many researchers in the world is focused on the study of problems related to the prevention of mycotoxin contamination of food and pharmaceutical products and animal feed. To date, a significant progress has already been made in this direction: the structures of mycotoxins were established [1], their mechanisms of actions were revealed [1,2], methods of their detection and identification were elaborated [3,4,5,6], and approaches to their detoxification were developed inside of various materials [7,8,9] and in vivo (in the body of animals) [10]. However, large volumes of organic wastes containing mycotoxins, in particular coming from agriculture, compose new tasks related to the need to solve environmental problems. As human civilization develops, these problems require more sharply focused attention than before. Now, on the one hand, there is an increase in people’s needs for agricultural products, and on the other hand, there is an increase in the requirements for the sustainable development of various fields of industry and the economy as a whole.

The growth of the world’s population and the large-scale consumption of poultry meat and eggs has led to the emergence of a large number of poultry wastes (hatchery wastes, birds’ excrements, poultry drops, and litters), which require rational safe processing or disposal [11,12]. This problem is gradually covering many regions of the world today.

Recently, anaerobic digestion (AD) was recognized as one of the promising approaches to the conversion of these multi-tonnage wastes as a renewable source of raw materials into a fuel in the form of biogas with high methane content [12,13,14]. Digestate obtained after AD can be used as a soil fertilizer [15,16]. Despite all the prospects of AD of poultry manure, there are a number of urgent problems limiting its wide application. In particular, poultry waste loaded to the methantank, as well as AD digestate obtained at the exit from this process, may contain not only antibiotic residues, antimicrobial resistance genes, hormones, and heavy metals, but also mycotoxins [13,15,17]. Residual amounts of the pollutants negatively affect the functional activity of cells-biocatalysts of methanogenesis (anaerobic sludge), which limits its possible long-term use in methanogenesis, decreases the yield of biogas [18], and reduces the potential of practical applicability of the digestate for improving soil quality.

One of the ways for overcoming the problem is pretreatment of poultry manure before its loading into the methantank or during AD [13,19]. The various methods used for the preprocessing of poultry manure can be conditionally divided into physical, chemical, physical–chemical, and biological [20]. Activated carbon, carbon cloth, biochar, magnetic addition, thermal and electrolysis treatment, etc., are used to extract various micropollutants from the litter [13,21]. 

Among the disadvantages of using sorbents, it is possible to mention the extraction of micropollutants from the litter; it does not ensure decomposition and detoxification of mycotoxins. It is relevant to search for approaches to pretreatment of manure, ensuring the destruction of micropollutants, limiting the effective AD of poultry manure, and subsequent environmentally safe use of digestate. It was noted that when developing approaches to cleaning litter from micropollutants, the main attention of researchers today is focused on the antibiotics, antimicrobial resistance genes, and hormones [13]. Therefore, in this review, we decided to focus on the issues of cleaning poultry manure from mycotoxins within its methanogenic treatment.

**Figure 1:**
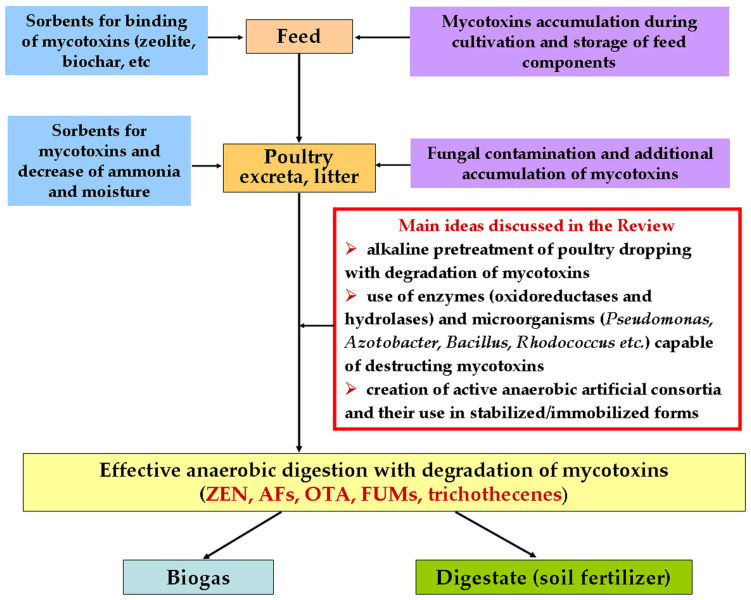
General scheme of main points discussed in this review concerning the problems of mycotoxins’ presence in poultry wastes and main ideas aimed at possible solutions of the problems in the frame of methanogenesis.

It should be emphasized that mycotoxins in the litter can have two main origins (Figure 1): (i) mycotoxins can accumulate in the feed during the cultivation, harvesting, transportation, and storage of the feedstock [22] and (ii) mycotoxins appear as a result of litter contamination by microscopic fungi during its transportation and storage due to high content of proteins, moisture, etc. [23]. It means that we always have mixtures of mycotoxins in the poultry manure loaded to methantanks. Moreover, in some cases, we have the mycotoxins both in free and sorbed (on/in various sorbents) forms.

It is known that anaerobic sludge has the ability to biodegrade mycotoxins during methanogenesis. The 12–99% decrease in the content of several mycotoxins under mesophilic and thermophilic conditions was recorded both in batch and in semi-continuous reactors. However, only the presence of individual mycotoxins in relatively low concentrations in the litter loaded to AD made it possible to overcome the difficulties with these micropollutants [24].

A negative effect of aflatoxin B1 (possessing a high enough toxicity) at its concentration above 100 µg/kg of wet litter mass on the AD was revealed and residues of the mycotoxin were noted in the resulting digestate [18]. Of cause, if a mixture of mycotoxins is present in the litter, the situation may worsen. Nowadays, it is known that manure introduced into the soil ranks third among the sources of mycotoxins after plant residues and rain washes [25,26]. Herbs fertilized with manure contain more mycotoxins than herbs fertilized with mineral enrichers [27,28]. 

Therefore, when developing effective approaches to the processing of bird droppings by AD, it is necessary to take into account all mentioned facts, since its processed residues, as well as manure, are introduced into the soil as fertilizers. Before using digestate in agriculture, it is important to ensure the absence of mycotoxins there [18].

Electron irradiation, cold atmospheric plasma, pulsed electric fields, ozonation, and enzymatic hydrolysis are among the most promising innovative methods of detoxification of foods and feeds from aflatoxins (AFs), ochratoxin A (OTA), fumonisins (FUMs), zearalenone (ZEN), and trichothecenes [10,29,30,31,32]. However, from an economic point of view, it is impractical to use these approaches for the decomposition of mycotoxins in the litter.

Alkaline pretreatment of bird droppings before AD is a well-known, affordable, and effective approach, which today is mainly used to increase the efficiency of methanogenesis by reducing the amount of solid components in it and the concentration of nitrogen-containing components that inhibit methanogenesis [19]. 

In this review, for the first time, alkaline chemical pretreatment of bird droppings is discussed as an approach aimed at the transferring of mycotoxins inside bird droppings into a liquid fraction from sorbents and their possible partial or complete destruction before introducing the litter into the methantank for AD (Figure 1).

In this review, we focused on the specific problems of bird droppings containing mycotoxins and approaches to solving them for effective AD, which allows, in addition to obtaining biogas, to obtain digestate that does not pollute the environment with mycotoxins when it is applied to the soil as a fertilizer. The review analyzes the recent developments of current biotechnology and synthetic biology, which can be applied to improve the characteristics and to intensify the processes under consideration.

## 2. Mycotoxins in Poultry Manure

It was found that most of the mycotoxins present in the feed are excreted with droppings in birds [33,34,35]. Recently, there have been significantly fewer cases of consumption of large amounts of mycotoxins by birds than 10–20 years ago, since strict quality control of feed is organized in developed countries. However, the problems of animal consumption of mycotoxin-contaminated feed remain relevant for some countries of the African continent [36] and the Asian region [37]. Locally produced feed is often infected with mycotoxins [38].

The following mycotoxins are among those revealed in various feeds: AFs (AFB1, AFB2, AFG1, AFG2, AFM1), OTA, ZEN, FUMs (FB1, FB2, FB3), patulin (PAT), and trichothecenes, such as deoxynivalenol (DON) and T-2 toxin (T-2) [29,39,40,41].

We analyzed the information about the mycotoxins detected in the different samples of litter and collected it in Table 1 [33,35,42,43,44,45,46]. Apparently, from the entire known list, ZEN, AFs, and DON require increased attention when developing approaches to cleaning bird droppings from these mycotoxins.

An analysis of the collected results obtained by various researchers (Table 1) showed that in some cases the concentration of mycotoxins in leachates of broiler chickens may be over 50% of the concentration that enters the animal body with the feed.

It should be noted that mycotoxins can be included in bird droppings not only in free form, but also in the matrix of sorbents that enter the litter in different ways [47,48,49,50,51,52,53,54]. Bird droppings almost always contain sorbents, since they are used as a feed additive, introduced into the litter for sorption of droppings together with excess moisture to reduce the growth rate of mycelial fungi [47].

The manure is also poured with sorbents in layers to sorb the released ammonia (Table 2) [48,49,50,51,52,53,54]. Interestingly, the accumulation of ammonia inside sorbents with mycotoxins can probably catalyze the partial degradation of these toxic compounds under conditions of increasing alkalinity. Next, we will specifically discuss the effect of the alkaline pH of media with mycotoxins on their destruction.

If chickens consume feed additives with sorbents, then mycotoxins associated with the sorbent are excreted from the bird’s body as part of bird droppings. Most of the sorbents used are most often of natural origin and do not pose a threat to ecosystems by themselves [55]. However, mycotoxins sorbed on/in them, due to their possible subsequent desorption, pose a direct threat to living organisms and can accumulate in soil and plants [56].

Thus, when developing approaches to cleaning bird droppings from mycotoxins, it is required not only to consider direct ways of destruction of toxic molecules, but also to take into account the presence and properties of sorbents in relation to mycotoxins of different nature that may appear in the composition of poultry manure by different ways (Table 2).

## 3. Prospects and Features of the Process of Detoxification of Mycotoxins in the Litter by Alkaline Pretreatment

To increase the efficiency of the methanogenic conversion of bird droppings into biogas, promising is the use of hydrolytic pretreatment for the degradation of macrobiomolecules (proteins, fats, carbohydrates, nucleic acids, etc.) to components with a lower molecular weight, which, when ingested in the methantank, can undergo biotransformation at a higher rate [19]. At the present stage of studying the methanogenic treatment of manure, the main attention of researchers is focused on the hydrolysis of the residues of lignocellulose components contained in it [57]. This is dictated by the fact that their content can reach 38–40% of the dry matter mass [58]. 

It is known that the preliminary alkaline treatment of manure with the addition of 3% (*w/w*) NaOH increases methane yield by 143.5 and 180.2% under mesophilic and thermophilic conditions, respectively [59]. Alkaline pretreatment (0.3 g of NaOH/g of substrate) is also recognized as effective in the methanogenesis of poultry waste [58,60].

Chemical pretreatment at alkaline pH values looks attractive when assessing the possibilities of simultaneous destruction of these macromolecules and detoxification of mycotoxins in the litter before loading into the methantank. Alkaline pretreatment of animal waste before methanogenesis is generally a well-known and fairly common approach, while it has been shown that substrates with a pH above 6.0 are more preferable for AD [60,61].

The use of various alkaline chemicals, including ammonium, limewater, sodium hydroxide, potassium hydroxide, and sodium carbonate, was studied in the processes of destruction of mycotoxins or their neutralization in solid foods and feed [62,63]. It is known that mycotoxins are more resistant to degradation in an acidic environment than under alkaline conditions [28,61].

Analysis of the literature data showed that alkaline pretreatment of various raw materials can contribute to the complete or partial detoxification of mycotoxins (such as ZEN, AFs, DON, FUMs, PAT, and OTA) contained in them (Table 3) [46,63,64,65,66,67,68,69,70,71,72,73]. Based on the data, it is possible to suggest the appropriate use of alkaline pretreatment of bird droppings to reduce or eliminate mycotoxins in this raw material.

The study of the products formed as a result of alkaline treatment of mycotoxins was carried out [40,62,63,65,66,69,70,73,74,75,76]. ZEN contains a resorcylic acid framework associated with a 14-membered macrolactone fragment, the stability of which is reduced under certain conditions. When ZEN was heated with NaOH, its decarboxylation occurred and formation of a low-toxic alkylresorcinol product of 1-(2,4-dihydroxyphenyl)-10′-hydroxy-1′-undecen-6′-one was determined [69].

In a medium with a high pH (in the presence of Ca(OH)_2_ or NH_4_OH), the lactone ring of AFs was opened and brought a decrease in the toxicity of the media [70].

The action of the ammonia solution on AFB1 led to the formation of two main decomposition products that retain the difuran part but lose the lactone ring: AFD1 and AFD2. The detoxification efficiency of the AFs in an alkaline media depends on the temperature, pressure, humidity, and duration of the treatment [74].

An epoxide in the structure of DON is essential for its toxicity, but it can be destructed under alkaline conditions [62]. According to the peaks and molecular ions detected in the chromatograms of degraded samples, possible chemical reactions between DON and NH_3_ solution leading to mycotoxin detoxification were identified [66].

It was found that PHFB1, HFB1, PHFB2, and HFB2 predominate among hydrolytic products during alkaline treatment of FB1 and FB2 [63,70].

PAT is successfully decomposed under alkaline conditions in the presence of ammonium hydroxide when the medium is heated [71]. A decrease in the temperature of the reaction medium while maintaining alkaline conditions, e.g., by the presence of CaCO_3_, leads to a noticeable decrease in the degree of PAT hydrolysis [72].

The opening of the ring in the structure of OTA during its alkaline treatment was also shown [75]. The most successful degradation of OTA is observed at pH above 10 and is described by first-order kinetics [76]. It was proved that alkaline medium creates more favorable conditions for extraction of OTA from solid carriers. Transferring of OTA to a liquid phase and destruction was revealed under alkaline conditions with greater efficiency compared to media with acidic pH values [73].

If we take into account that mycotoxins (PAT, DON, NIV, FB1, and FB2) present mainly in the sorbent-bound form in bird droppings (Table 2), then we can expect that alkaline pretreatment can contribute to desorption and transition of the mycotoxins to the reaction medium due to their fairly good solubility in the aqueous phase [74]. Among mycotoxins, DON and FUMs should be expected among those that can be present in the litter and decomposed under alkaline conditions.

Thus, alkaline pretreatment can be seriously taken into account for processing poultry manure not only to increase the rate of accumulation of biogas as a result of pre-hydrolysis of macromolecules in the content of the used substrates for AD. Additionally, in order to maximize the efficiency of decomposition of mycotoxins present in the treated raw materials, the alkaline medium seems to be effective. However, when discussing the alkaline treatment of mycotoxins in poultry manure, it should be taken into account that it contains about 30% of protein (in relation to total dry substances) [58]. It is known that during alkaline hydrolysis of protein and mycotoxins, the accumulation of free amino acids and organic acids occurs in the hydrolysate of manure [72]. Formation of dehydro- and cross-linked amino acids, such as dehydroalanine, methyldehydroalanine, beta-aminoalanine, lysinoalanine, ornithinoalanine, histidinoalanine, phenylethylaminoalanine, lanthionine, and methyl-lanthionine, and other by-products may occur [77]. Racemization of L-amino acid isomers to D-analogues with low biological digestibility was also revealed. Some of the resulting products (such as lysinoalanine and lanthionine) are toxic to humans and animals [78]. There is no information regarding the effect of these compounds on anaerobic digestion, probably due to the fact that the content of lysine and alanine in the litter is relatively low. If these compounds enter the soil together with anaerobic digest after the AD of the litter, significant negative toxic effects in relation to living organisms probably should not be expected. Since different products are formed during the alkaline transformation of proteins that can enter into various interactions with other hydrolytic products, e.g., coming from the destruction of different mycotoxins, this situation should be checked separately.

## 4. Enzymes as Destructors of Mycotoxins in Poultry Manure under Conditions of Methanogenesis

The possible destruction of mycotoxins directly in AD under the action of anaerobic biocatalysts of methanogenesis should be taken into account in the case of incomplete detoxification of mycotoxins contained in bird droppings at the stage of its alkaline pretreatment. It is known that the destruction of mycotoxins can be carried out by a number of microorganisms involved in AD when various polluted substrates are used in the process [18,24,79,80,81,82,83,84,85]. The success of their effect on mycotoxins is due to the fact that they have a number of enzymes capable of catalyzing the transformation of mycotoxins (Table 4) [81,82,83,84,85]. 

Today, numerous enzymes, mainly belonging to the class of oxidoreductases and hydrolases, are known to modify various mycotoxins [31]. It is noteworthy that several enzymes degrading AFs (i.e., F_420_-dependent reductase *Ms*FDR), DON (i.e., aldo-keto reductase *Ssp*AKR18A1), FUMs (i.e., esterase *Sm*FumD), and ZEN (i.e., esterase *Cr*ZHD and peroxiredoxin) have been reviewed recently [31].

**Figure 2:**
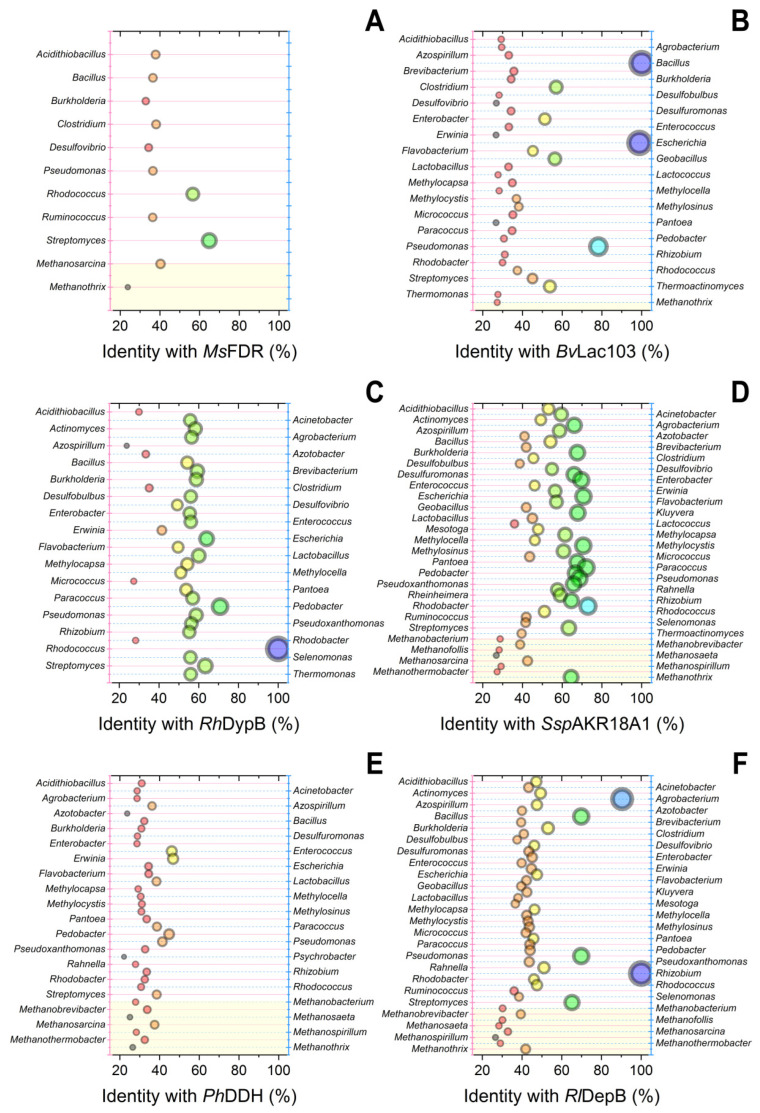
Bubble plot of homologous enzymes being catalytically active towards AFs (**A**–**C**) and DON (**D**–**F**) screened within known bacteria and archaea present in sludge microbial consortia. Protein BLAST (https://blast.ncbi.nlm.nih.gov/, accessed on 15 January 2023) was performed using (**A**) F_420_-dependent reductase *Ms*FDR from *Mycolicibacterium smegmatis* (PDB 3F7E); (**B**) laccase *Bv*Lac103 from *Bacillus vallismortis* (GenBank AGR50961.1); (**C**) peroxidase *Rh*DypB from *Rhodococcus jostii* (GenBank AYJ72200.1); (**D**) aldo-keto reductase *Ssp*AKR18A1 from *Sphingomonas* sp. (GenBank ASY03293.1); (**E**) PQQ-dependent reductase *Ph*DDH from *Pelagibacterium halotolerans* (GenBank QJR20540.1); (**F**) aldo-keto reductase *Rl*DepB from *Rhizobium leguminosarum* (PDB 7UTF). The best samples are presented. The size and color of bubbles corresponds to the certain percent of identity of amino acid sequence to the enzyme selected for the analysis (horizontal axis).

**Figure 3:**
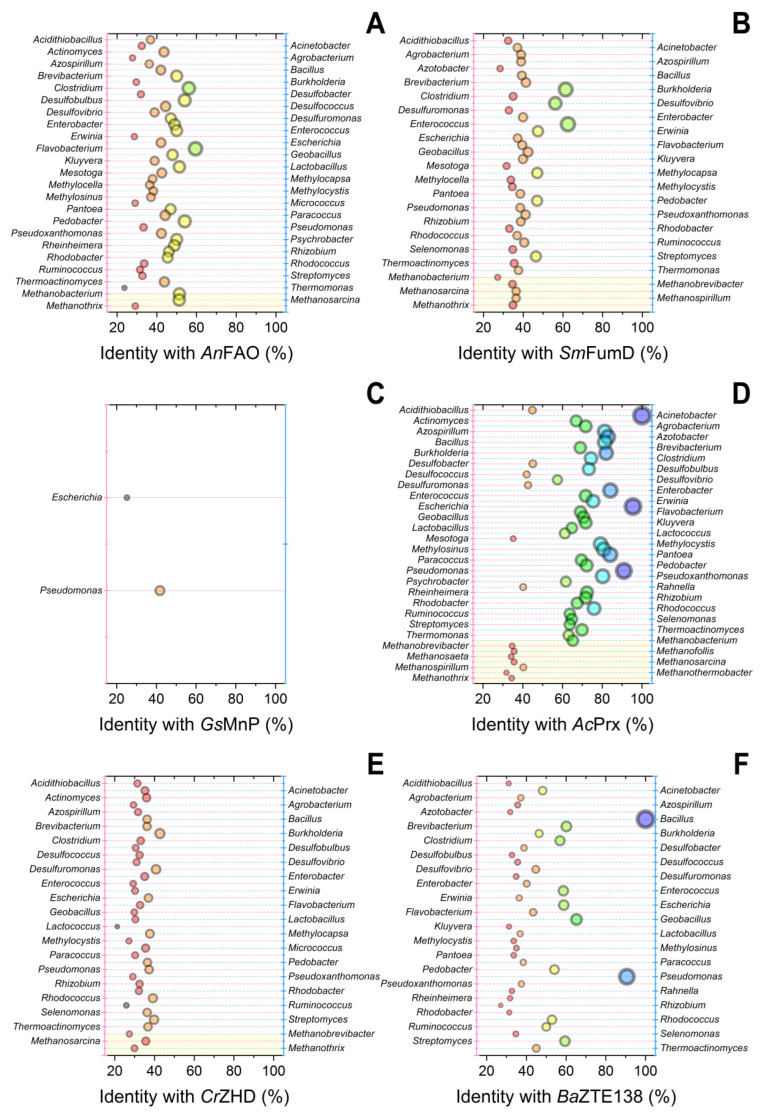
Bubble plot of homologous enzymes being catalytically active towards FUMs (**A**–**C**) and ZEN (**D**–**F**) screened within known bacteria and archaea present in sludge microbial consortia. Protein BLAST (https://blast.ncbi.nlm.nih.gov/, accessed on 15 January 2023) was performed using (**A**) amine oxidase *An*FAO from *Aspergillus niger* (GenBank GKZ63144.1); (**B**) esterase *Sm*FumD from *Sphingopyxis macrogoltabida* (GenBank D2D3B6); (**C**) Mn-dependent peroxidase *Gs*MnP from *Gelatoporia subvermispora* (GenBank AUR34180.1); (**D**) peroxiredoxin *Ac*Prx from *Acinetobacter calcoaceticus* (GenBank MBP2602639.1); (**E**) esterase *Cr*ZHD from *Clonostachys rosea* (PDB 3WZL); (**F**) thioesterase *Ba*ZTE138 from *Bacillus amyloliquefaciens* (GenBank WP_017417881.1). The best samples are presented. The size and color of bubbles corresponds to the certain percent of identity of amino acid sequence to the enzyme selected for the analysis (horizontal axis).

Specifically for this review, known amino acid sequences of enzymes capable of destructing the mycotoxins were searched within microorganisms whose presence has been established in consortia functioning in AD processes [86]. For that, the GenBank database was screened on non-redundant protein sequences of known degraders using Protein BLAST (https://blast.ncbi.nlm.nih.gov/, accessed on 15 January 2023) (Figure 2 and Figure 3).

To improve representative sampling before screening in anaerobic microbial consortia, the number of enzymes destructing various mycotoxins was enlarged by laccase *Bv*Lac103 and peroxidase *Rh*DypB both acting on AFs [87,88], by PQQ-dependent reductase *Ph*DDH and aldo-keto reductase *Rl*DepB both modifying DON [89,90], by amine oxidase *An*FAO and Mn-dependent peroxidase *Gs*MnP both degrading FUMs [91,92], and by novel thioesterase *Ba*ZTE138 hydrolyzing ZEN [93]. Initially, there was a bias towards the pre-selection of these enzymes precisely inside the microorganisms composing different methanogenic microbial consortia. For this purpose, information about the participants of 54 genera that catalyze AD processes summarized previously [94] was used in the current analysis. In total, the estimations of 12 enzymes capable of detoxifying 4 main types of mycotoxins (AFs, DON, FUMs, and ZEN) were undertaken in these microorganisms (Figure 2 and Figure 3).

Thus, it was not surprising that source bacteria, e.g., *Bacillus* producing laccase *Bv*Lac103 and thioesterase *Ba*ZTE138 (Figure 2B and Figure 3F), *Rhodococcus* producing peroxidase *Rh*DypB (Figure 2C), *Rhizobium* producing oxidoreductase *Rl*DepB (Figure 2F), and *Acinetobacter* producing peroxiredoxin *Ac*Prx (Figure 3D), had the highest scores. Nevertheless, several other potent bacteria could be highlighted also, namely *Pseudomonas* which could possess homologous aflatoxin-degrading laccase *Bv*Lac103, zearalenone-detoxifying peroxiredoxin *Ac*Prx, and thioesterase *Ba*ZTE138; *Escherichia* which could synthesize highly homologous aflatoxin-degrading laccase *Bv*Lac103 and zearalenone-detoxifying peroxiredoxin *Ac*Prx; and *Agrobacterium* which could produce homologous deoxynivalenol-modifying oxidoreductase *Rl*DepB.

It is noteworthy that peroxiredoxin *Ac*Prx being of bacterial origin had the highest diversity of high score homologues among different bacteria. Meanwhile, enzymes of fungal origin (e.g., *Gs*MnP, *Cr*ZHD, and, to a lesser degree, *An*FAO) had the lowest median scores. There are a number of fungal cells in methanogenic consortia [94], but they were not considered in the current work. Anyway, when estimating a degradability of the current four mycotoxins, the worst capability to be degraded by the methanogenic consortia of microorganisms was in the case of FUMs. This is curious information since FUMs are the least toxic and have chemical structures being close to usual lipids.

Possibly, there are gaps in our knowledge of FUMs degradation pathways in sludge and these microorganisms possess their own alternative FUMs-degrading enzymes not described so far. However, the results of the analysis of enzymatic activities of the microbial participants of AD processes completely correlated with the data shown in Table 4, where the degradation of FUMs is low (15%) as compared to AFs.

Even if the residual concentrations of FUMs detected in excreta may be overestimated by 15–19% [95], the destruction of these mycotoxins by enzymes present in the cells of methanogenic consortia is clearly insufficient. Moreover, conversion degree of FUMs can be up to 67% (Table 3), with alkaline pretreatment of substrates containing mycotoxins before their loading to AD. 

It should be noted that a significant part of enzymes that are present simultaneously in two comparable categories (catalyzing the transformation of mycotoxins and ensuring the metabolic activity of cells inside of the methanogenic community as well as the consortia of the cells present in the poultry excreta [96]) exhibit their functional activity at alkaline pH values (7.6–8.2), which are more often typical for bird droppings. Thereupon, it seems that the conversion degree of FUMs can be totally improved.

Moreover, mycotoxin-degrading enzymes that are synthesized and secreted by cells can be partially inactivated during AD or composting. This is due to the formation of non-covalent complexes of these enzymes (most often hydrolases) with humic substances formed in reaction media during degradation of organic matter under the action of anaerobic consortia [97].

It has been shown that the higher the concentration of humic substances present in the reaction medium, the more significant the inhibition of the metabolic activity of cells of anaerobic consortia [98].

In addition, some mycotoxins may become less accessible for enzymatic degradation in AD as a result of humification of the initial organic substances under anaerobic conditions. Humic substances can easily bind different mycotoxins [99,100,101]. It should be reiterated here that alkaline conditions are favorable for the transformation of humic acids into their soluble forms. This means that they can contribute to the “release” of mycotoxins from such bound state and present them in bioavailable state for enzymatic conversion.

The targeted introduction of the necessary enzymes to AD can be considered as one of the possible options for improving the conversion by compensating for the lack of enzymes capable of catalyzing the detoxification of mycotoxins in reaction media during AD of poultry excreta. However, the use of enzymes capable of destroying several mycotoxins at once appeared to be the most effective approach [10,102]. In addition, the introduction of enzymes into AD is most appropriate in a stabilized form, possibly as an immobilized protein in sorbents used both at the stage of manure storage and at the stage of AD (Figure 1, Table 2). To date, such examples were not found in the literature.

## 5. Expected Potential in Development of Anaerobic Biocatalysts for AD of Poultry Manure Contaminated with Mycotoxins

AD is often proposed as a way of processing organic waste contaminated with mycotoxins to obtain the final products (biogas and digestate) (Figure 1). In this regard, there are studies evaluating the effect of mycotoxins on the process of methanogenesis in various modes of the process [79]. The results of these studies have demonstrated that the implementation of such processes is possible. The decomposition of mycotoxins significantly depends on their initial concentration in the medium, the temperature in the methantank, as well as the retention time [79]. Considering that methanogenesis, as a rule, is a long process (Table 4), it is possible to overcome the inhibition of anaerobic consortium by mycotoxins during the retention of the substrate with toxins in the reactor.

At the same time, the process of transformation of substrates containing mycotoxins in methantank is characterized by a number of features, among which one can note a decrease in the microbiological diversity of consortia, the transformation of mycotoxins into their various derivatives, and incomplete transformation of mycotoxins [18,24,79,81,103]. In this regard, there is an obvious interest in various solutions that make it possible to reduce or neutralize the toxic effects of components of bird droppings on the methanogenic consortia.

There are several possible solutions: an improvement of anaerobic consortia content by the introduction of new microbial components to them; a quantitative increase in cells already present in the consortia and producing enzymes destructing various mycotoxins; and an application of immobilized forms of consortia to stabilize their functioning in AD. Of course, the composition of the anaerobic consortium used for AD of poultry manure can be improved by introducing cells capable of detoxifying mycotoxins into the microbial community. The choice of such cells can be made on the basis of the search for genes responsible for the synthesis of the necessary enzymes, taking into account the fact that they are able to carry out functioning under anaerobic conditions.

In several recent investigations, new information about mycotoxin destructors, some of which are facultative anaerobes, was published [104,105]. However, not all cells from the published list can be included in consortia. For example, mycelial fungi capable of synthesizing enzymes that hydrolyze some mycotoxins are producers of other mycotoxins. Bacterial cells of the genus *Bacillus*, according to Figure 2B and Figure 3F and the published data [106], can exhibit a high activity against ZEN, Afs, and DON, but these cells are producers of substances with pronounced antimicrobial activity against participants of methanogenic consortia. Moreover, this antimicrobial activity is enhanced when these compounds originated from *Bacillus* cells are combined with individual enzymes hydrolyzing mycotoxins [94,107].

An interesting result for the development of new AD consortia may be obtained using microorganisms that were recently isolated from the intestinal microbiome of broilers, where they showed toxicity mitigation of some mycotoxins [108,109]. The weak alkaline pH in the intestine of broilers contributed not only to the manifestation of activity by those enzymes of intestinal microorganisms that can catalyze the destruction of mycotoxins, but also additionally could catalyze the alkaline degradation of the same toxic compounds.

It is possible not only to create artificial consortia by introducing new types of cells into natural sludge for the methanogenesis of poultry manure and mycotoxins contained in it, but also to artificially increase the proportion of those bacteria that exhibit catalytic activity against mycotoxins. For example, *Pseudomonas* and *Rhodococcus* genera cells are widely represented in anaerobic consortia, which, as it turned out, have the genetic potential to synthesize enzymes that catalyze the destruction of ZEN (Figure 3). At the same time, there is a positive experience of creating effective anaerobic consortia based on these Gram-positive and Gram-negative cells of the genus *Rhodococcus* and the genus *Pseudomonas*, respectively, which enhances the effect of each other, and their use is successful as a part of artificial consortia in combination with anaerobic sludge [110,111].

Currently, as noted above, the maximum difficulty is caused by the detoxification of FUMs. Therefore, it is advisable to increase the number of microbial cells in synthetic consortia that are able to catalyze the corresponding reactions. *Pseudomonas* bacterial cells possess the ability to destroy FUMs, which was established not only during theoretical analysis (Figure 3C) but was also confirmed experimentally [112].

For cells of the genus *Azotobacter*, which are present in many methanogenic consortia (Figure 2 and Figure 3), the activity against FUMs has been shown in a number of studies [113,114]. Probably, an increase in the number of these cells as components of improved variants of microbial communities can be considered for the destruction of mycotoxins in bird droppings within AD with the hope of success.

It is obvious that the preservation of the introduced changes in anaerobic consortia (in terms of composition or number of cells) is an important condition for the successful processing of raw materials and mycotoxins contained therein. Successful application of various methods of immobilization of methanogenic consortia has been shown to maintain stable functioning of such biosystems [14,115,116]. Simultaneously, it was found that the introduction of an immobilized anaerobic consortium into the reactor really reduces the inhibitory effect of many negative factors and reduces the period of reactor output to the operating mode associated with the rapid adaptation of cells to different substrates due to the implementation of quorum processes.

The application of the sorption as a method of cell immobilization resulted in gradual desorption of microorganisms from carriers during AD. This can lead to destabilization of the functioning of cells. Moreover, the use of carriers based on natural polymers leads to their gradual chemical destruction and physical degradation, including deformation under the pressure of various gas metabolites of anaerobic cells accumulating inside the carriers during methanogenesis [115]. However, various mineral sorbents introduced into the substrates for AD under discussion (Figure 1), due to their objective application, can be considered as carriers for the immobilization of cells of anaerobic consortia.

It should be noted that today the successful application of various carbon-based functional materials for improvement of AD characteristics is shown [117]. In this regard, it seems appropriate to study the role of these materials not only in increasing the efficiency of methanogenic conversion of such a substrate as poultry manure and litter, but also in the destruction of various mycotoxins. Probably, someone will take this mentioned idea into realization. In the meantime, such materials are considered only as sorbents. 

Known processes based only on the physical–chemical destruction of mycotoxins are characterized by high enough rates and efficiency of degradation of mycotoxins (62–100% degradation for 0.25–18 h, Table 3). However, they can be accompanied by the accumulation of toxic intermediates. The degradation of mycotoxins using biological strategies is a promising solution to the problem of mycotoxins, since it can result in the formation of small amounts of toxic products or their complete absence [5,10]. The disadvantage of biocatalytic methods of mycotoxin degradation is a long period of necessary treatment (15–100% degradation is achieved within 25–60 days, Table 4). The use of hybrid physical–chemical and biocatalytic approaches for the destruction of micropollutants in animal wastes is aimed at ensuring an optimal combination of the advantages of each of the used methods of mycotoxin degradation and reducing the negative effect of their typical disadvantages [14]. There are still few studies in this direction, and therefore they are promising for development.

## 6. Conclusions

Bird droppings containing various mycotoxins are very complex substrates for anaerobic transformation without pretreatment and combination of certain catalytic conditions for its deep conversion. The analysis of the currently known information on the methods of implementing AD and approaches to the transformation of certain mycotoxins to safe media by using positive experience in the formation of stable artificial microbial consortia was undertaken. It gives ideas of the directions in which it makes sense to look for ways to intensify and improve the processes under discussion. The review made it possible to outline strategic directions for the effective implementation of methanogenesis and transformation of not only the main components of the poultry manure, but also the mycotoxins contained in it. Such information includes a certain scientific basis for the development of eco-friendly approaches to the treatment of bird droppings, the production of biogas, and non-toxic digestate acceptable for use as fertilizer. The hybrid chemical–biocatalytic approach to the transformation of bird droppings into biogas with preliminary alkaline treatment contributes to the sustainable development of the agricultural industry, reduces toxic effects on the environment, and protects the health of living organisms.

## Figures and Tables

**Table 1:** Mycotoxins in poultry excreta in the case of their presence in the feed.

Object of Analysis (Country) [Reference]	Mycotoxin	Concentration (μg/kg)	
		in Feed	in Excreta
Leachates of broiler chickens (France) [35]	ZEN	27 ± 9 400 ± 120	12 ± 6 270 ± 90
Manure of broiler chickens (China) [42]	ZEN + AFB1	58.6 ZEN + 14.5 AFB1	38.8 ZEN + 2.3 AFB1
Droppings of broiler chickens (Poland) [33]	AFB1	1000 5000	290 2740
Chicken excreta (Austria) [43]	AFs (AFB1, FB2, AFG1, AFG2, AFM1)	18 515	4 30
Leachates of broiler chickens (Spain) [44]	DON	5000 15,000	22.0 24.1
Chicken excreta (USA) [45]	T-2	3500	682
Fecal samples of rats and sheep (USA) [46]	FB1	rats 1000 sheep 50	530 6

**Table 2:** Examples confirming the widespread regular use of sorbents at different stages of the formation and further processing of bird droppings.

Sorbent (Country) [Reference]	Main Purpose of the Sorbent Application	Procedure of Sorbent Addition	Dose of Sorbent Introduction
Brown coal (Australia) [48]	Ammonia sorption	Introduction of sorbent into the litter for broilers	20% (*w/w*) of litter
Deodoric^®^ (a mixture of zeolite and perlite with six bacterial strains) (Poland) [49]	Reducing humidity, preventing the appearance of ammonia in the air	Introduction of sorbent into the litter for chickens	170 g/m^2^ of litter (once a week)
Natural Zeolite (China) [50]	Influence of the microbial society to reduce abundance of antibiotic resistance genes	Additives to compost with chicken manure	50 g/kg of wet chicken manure
Zeolite and bio coal (China) [51]	Decrease in variety of antibiotic resistance genes	Additives to compost with chicken leachates	Sorbent ZL (5% *w/w* zeolite), BC (5% *w/w* bio coal), or ZB (per 5% *w/w* of both zeolite and bio coal) in compost mass
Diatomite and bentonite (Iran) [54]	Influence on the microbial community in order to reduce spreading of antibiotic resistance genes	Additives to compost with poultry manure	5% (*w/w*) sorbent mixture (diatomite and bentonite) in composted mass
Biochar, bentonite, and zeolite (Australia) [53]	Influence on chemical and water-retaining properties of excrements and granulation characteristics of decomposed excrements	Additives to chicken feed	2% (*w/w*) biochar, 2% (*w/w*) zeolite, or 4% (*w/w*) biochar in the broiler feed
Synthetic polymer based on methacrylic acid (TMU95) with macroporous structure (Iran) [54]	Binding of AFs	Additives to feed for ducklings	5 g TMU95/kg diet when 200 µg of AFB1 is in 1 kg of feed

**Table 3:** Detoxification of mycotoxins under alkaline conditions.

Mycotoxin (Country) [Reference]	Object of Contamination	Process under Alkaline Conditions	Degradation
ZEN (China) [65]	Crude corn oil 1178.7 μg ZEN/kg	2 M NaOH, degumming	100% ZEN
AFs (AFB1, AFB2, AFG1 and AFG2) (European Union) [66]	Groundnut press cake (286 μg AFs/kg)	50 kg of the press cake, 0.6 L water, and 2 kg 25% *w/w* NH_4_OH were mixed for up to 3 h at a pressure of 10 kPa	95% AFG1 93% AFG2 85% AFB1 83% AFB2
AFs and FUM (Uganda) [67]	Maize	Soaking maize grains overnight in 1% slaked lime (Ca(OH)_2_) solution	Up to 90% AFs Up to 80% FUM
AFB1 (Mexico) [68]	Maize (125 µg AFB1/kg)	White maize (1 kg) was boiled for 45 min at 90 °C in 3 L of water with 10 g of lime (minimum content of Ca(OH)_2_ = 90%) and left to soak overnight (18 h at 24 °C, pH 10.2)	100% AFB1
DON (Spain) [69]	Wheat grains (2 mg DON/kg)	4.8% NH_4_OH, 90 °C, 2 h	Up to 75% DON
DON (China) [64]	Wheat grains (1 mg DON /kg)	Treatment of grains with alkaline electrolyzed water (AlkEW) (pH 9.5) at room temperature for 45 min	61.6% DON
FUMs: FB1 and FB2 (Germany) [63], (Italy) [70]	Maize (6480–8930 µg FUMs/kg)	0.33% or 1.67% lime solution, 90 °C, 15–60 min	Up to 68% FB1 and FB2
FB1 (USA) [46]	Model waste solution (10 mg/L)	1 L of 1 N KOH, 60 °C, 1 h	100% FB1
PAT (China) [71]	Model laboratory wastes (0.1–10 mg/L PAT)	1 mL of 5% ammonia was added to 100 mL of aqueous wastes with PAT and the resulting mixture was autoclaved at 120 °C for 15 min	99.9% PAT
PAT (China) [72]	Apple juice (1 mg/L PAT)	Treatment of juice by porcine pancreatic lipase immobilized on CaCO_3_ at 40 °C for 18 h	77.1% PAT
OTA (Japan) [73]	Model waste solution (100 mg/L)	0.1 M NaOH, 100 °C for 10 min	100% OTA

**Table 4:** Examples of mycotoxins’ degradation in AD.

Substrate for AD (Country) [Reference]	Mycotoxins and Conditions of AD	Degradation
Milled grain corn (75%) (Belgium) [81]	AFB1, ergot alkaloids (40 µg/L) OTA (50 mg/L) FB1, DON (400 mg/L) ZEN, T-2 (100 µg/L) 37 °C, 25 days	100% AFB1 100% ZEN 98% T-2 99% OTA 93% DON 70% FB1 67% ergot alkaloids
Cattle manure (45%), maize silage (45%), and maize flour (10%) (Italy) [82]	AFB1 (2–470 µg/kg) FUMs (115–3700 µg/kg) 38 °C, 50 days, pH 7.6–7.7	12–95% AFB1 15% FUMs
Corn grain (Italy) [83]	AFB1 (0.54–110 µg/kg) 37 °C, 60 days	69–87% AFB1
Corn (2.5%) and anaerobic sludge (97.5%) (1st medium) Corn (2.5%), pig slurry (22.5%), and anaerobic sludge (75%) (2nd medium) (Italy) [84]	AFB1 (100 µg/kg) 37 °C, 28 days, pH 7.4–7.6	69.7% AFB1 and 42% AFB1 in the 1st and 2nd medium, respectively
Corn (2%), pig slurry (40%), wood chips (40%), and cereal straw (8%) (Italy) [85]	AFB1 (100 µg/kg) 30–40 °C, 60 days	85.7% AFB1

## Data Availability

Not applicable.
